# Plasma Metabolomics in a Nonhuman Primate Model of Abdominal Radiation Exposure

**DOI:** 10.3390/metabo11080540

**Published:** 2021-08-13

**Authors:** Se-Ran Jun, Marjan Boerma, Zulema Udaondo, Sasha Richardson, Karla D. Thrall, Isabelle R. Miousse, John Seng, Rupak Pathak, Martin Hauer-Jensen

**Affiliations:** 1 Department of Biomedical Informatics, University of Arkansas for Medical Sciences, Little Rock, AR 72205, USA; zdominguez@uams.edu; 2 Division of Radiation Health, University of Arkansas for Medical Sciences, Little Rock, AR 72205, USA; mboerma@uams.edu (M.B.); jeseng@uams.edu (J.S.); rpathak@uams.edu (R.P.); mhjensen@uams.edu (M.H.-J.); 3 Department of Computer Science, Fayetteville State University, Fayetteville, NC 28301, USA; sfrichardson9@gmail.com; 4 Altasciences Preclinical Seattle, Everett, WA 98203, USA; kthrall@altasciences.com; 5 Department of Biochemistry and Molecular Biology, University of Arkansas for Medical Sciences, Little Rock, AR 72205, USA; iracinemiousse@uams.edu

**Keywords:** acute radiation syndrome, partial body exposure, nonhuman primates, metabolites, plasma

## Abstract

The acute radiation syndrome is defined in large part by radiation injury in the hematopoietic and gastrointestinal (GI) systems. To identify new pathways involved in radiation-induced GI injury, this study assessed dose- and time-dependent changes in plasma metabolites in a nonhuman primate model of whole abdominal irradiation. Male and female adult Rhesus monkeys were exposed to 6 MV photons to the abdomen at doses ranging between 8 and 14 Gy. At time points from 1 to 60 days after irradiation, plasma samples were collected and subjected to untargeted metabolomics. With the limited sample size of females, different discovery times after irradiation between males and females were observed in metabolomics pattern. Detailed analyses are restricted to only males for the discovery power. Radiation caused an increase in fatty acid oxidation and circulating levels of corticosteroids which may be an indication of physiological stress, and amino acids, indicative of a cellular repair response. The largest changes were observed at days 9 and 10 post-irradiation, with most returning to baseline at day 30. In addition, dysregulated metabolites involved in amino acid pathways, which might indicate changes in the microbiome, were detected. In conclusion, abdominal irradiation in a nonhuman primate model caused a plasma metabolome profile indicative of GI injury. These results point to pathways that may be targeted for intervention or used as early indicators of GI radiation injury. Moreover, our results suggest that effects are sex-specific and that interventions may need to be tailored accordingly.

## 1. Introduction

Acute radiation syndrome (ARS) is caused by total or partial body exposure to high doses of ionizing radiation, generally over a relatively short period of time (seconds to minutes). There are four subtypes of ARS: hematopoietic, gastrointestinal, cutaneous, and neurovascular [1,2]. Exposed individuals typically present within 24 h with nausea, vomiting, skin rashes, and confusion. A decrease in lymphocyte counts can be observed as early as 8 h after exposure. Death results from multi-organ failure, including systemic inflammatory response syndrome and life-threatening infections secondary to bone marrow failure [2]. Existing treatments include antimicrobial agents to control infections, cytokines to stimulate hematopoiesis, blood transfusions, and in some cases, bone marrow transplantation [1]. Despite these measures, most victims of high dose (>5.5 Gy) irradiation succumb within 3 weeks of exposure. In addition, approximately 50% of victims receiving 3.5 to 5.5 Gy does not survive past 2 months [1]. The development of more effective medical countermeasures against radiation exposure requires well characterized animal models that are as close as possible to humans, such as nonhuman primates (NHPs) [3]. To date, development of countermeasures targeting amelioration of gastrointestinal (GI) ARS has been limited by a lack of understanding of the natural history and disease sequelae of this syndrome in a large animal model [4].

We developed a GI-specific ARS model in male and female Rhesus monkeys (*Macaca mulatta)* that received abdominal irradiation at single doses of 8 Gy, 11 Gy, 12.5 Gy, and 14 Gy using 6 MV photons. In these NHPs, small intestine radiation injury was seen in the form of a reduced mucosal surface area, crypt depth, and villus height [5]. Animals were monitored up to 60 days after irradiation, when intestinal damage was still evident. To understand the metabolic pathways involved in the response to radiation, plasma samples were obtained from days 9 to 60 and subjected to metabolomics to obtain insights in molecular responses in GI ARS. Univariate comparisons within each radiation dose group and within each time point group were performed to identify metabolites that were significantly dysregulated in each radiation dose and over time compared to the sham group. To our knowledge, this is the first report on plasma metabolomics in an NHP model of abdominal irradiation. Our study may point to novel targets for medical countermeasures and aid in the development of predictive biodosimetry assays for delayed effects of acute radiation exposure.

## 2. Results and Discussion

### 2.1. Sex-Specific Patterns in Response to Abdominal Irradiation Were Observed in the Plasma Metabolomics Data

Sex is a confounding variable that must be taken into accounts for metabolite biomarker discovery and validation. Sex differences in plasma metabolomics data have been reported [6]. However, the effects of sex on the response to partial radiation exposure in NHP models remain understudied. First, we examined the sex-specific pattern of changes in metabolomic profiles across radiation doses and time points through principal component analysis (Figure S1). No pattern of significant differences between female (Figure S1A) and male (Figure S1B) groups was observed in baseline metabolomic profiles at 1 and 5 days prior to irradiation (D-5/D-1 (time point 1 (TP1) in Table 1) for all treatment groups. At days 9 and 10 after irradiation (D9/D10 (TP2) in Table 1), female and male groups showed similar patterns, where samples at TP2 diverged from samples at TP1. Interestingly, at D28/D29 (TP3 in Table 1) and D59/D60 (TP4 in Table 1), female plasma metabolomic profiles returned to their initial state, whereas male plasma metabolomic profiles remained different from baseline. Principle component analysis (PCA) revealed that female and male NHPs might have different recovery times in response to radiation (Figure S1). However, we did not have enough female samples in the experimental groups for a robust statistical analysis (Table 1). Therefore, for remaining statistical analyses, we considered only the male samples.

### 2.2. Radiation Dose-Dependent and Time-Dependent Metabolic Alterations in the Plasma Metabolomics Data Were Observed

**Figure 1:**
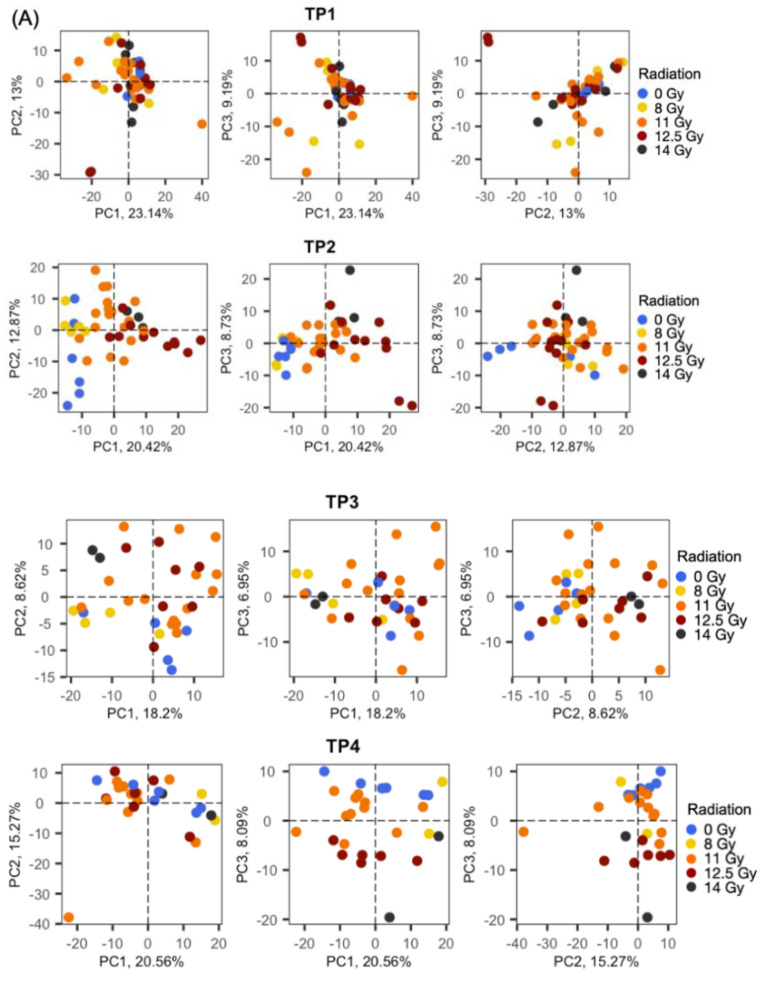

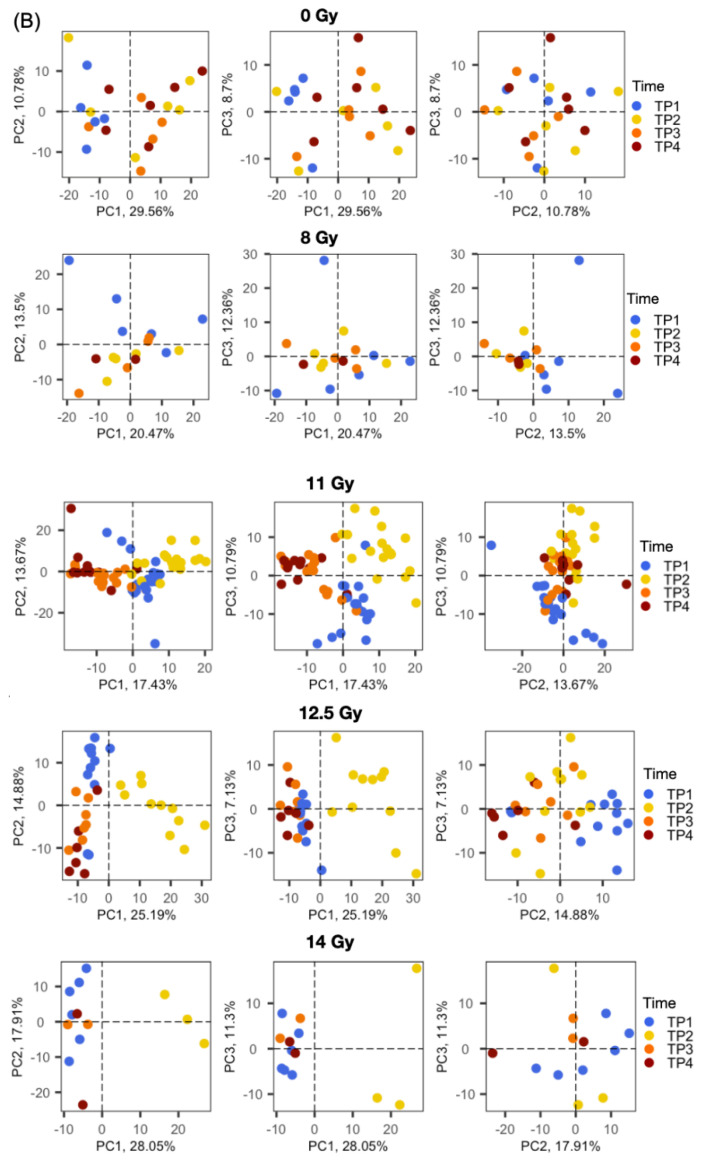
PCA plots. (**A**) comparison of radiation doses at different time points and (**B**) comparison of time points at different radiation doses. Overall, the highest segregation was observed for dose group 12.5 Gy and time group TP2. Note that only male samples were included in this PCA analysis. PC, principal component.

We performed PCA analysis of only male samples for each given time point (Figure 1A), and for each radiation dose (Figure 1B). As expected, no segregation between different radiation treatment groups was observed before radiation (TP1). After radiation exposure, PCA analysis revealed that all irradiated groups were well segregated as doses increased. The comparison between the closest time points before and after irradiation (TP1 and TP2) showed the largest variation, demonstrating that radiation exposure indeed induced metabolomic changes. Next, a PCA of the global metabolite profiles showed segregation between before irradiation (TP1) and after irradiation (TP2, TP3, TP4) for all radiation doses. Even the sham group presented noticeable metabolic changes between the various time points. Therefore, time varying confounders can lead to bias in metabolite biomarker discovery, and adjusting for time is necessary. We observed that segregation between before and after irradiation was clearer as radiation doses increased, demonstrating a dose-dependent effect of irradiation at the metabolome level. Samples in the TP2 group (9 and 10 post-irradiated days) were the most separated from TP1 (pre-irradiated days) group. Interestingly, segregation between TP2, TP3, and TP4 was also clearer as radiation dose increased. However, the 14 Gy group did not have enough samples to justify segregation in those comparisons. In short, plasma metabolomics data in NHPs showed dose-dependent and time-dependent metabolic changes in response to abdominal irradiation (Figure 1).

### 2.3. Global Metabolomic Profiles Identify Radiation Dose-Dependent and Time-Dependent Biochemical Signatures

The present study identified 696 compounds of known identity in this plasma sample set. Following volume-based normalization, imputation of missing values with the minimum observed value, and log transformation, we performed analysis of variance (ANOVA) and calculated fold change to identify biochemicals that are significantly different between experimental groups. Table 2 used fold change >2 and a significance level of *p* < 0.05 or a significance level of Benjamini–Hochberg (BH)-adjusted *p* < 0.05. Univariate analysis results and multivariate PCA results showed similar trends as a whole. Samples obtained before irradiation (TP1), as would be predicted, showed few differences between the various groups that were eventually given sham, low, or high dose radiation. This indicates that the groups had similar metabolomic profiles prior to dosing. The comparison between the closest time points before and after exposure showed the largest number of changes, demonstrating that radiation exposure does indeed cause detectable metabolomic changes, and that the sham treatment had minimal effects. Interestingly, fewest changes were observed over time after 8 Gy exposure. The dose of 12.5 Gy showed the largest changes at TP2, which decreased to some extent over time. This suggests the dosing regimen and response caused detectable metabolic changes, potentially reflecting a restoration of organismal homeostasis in surviving animals by the end of the experiment. However, metabolic alterations after 11 Gy were not restored over the 60day time course of the study, suggesting that high doses of radiation could have long-term effects on the metabolome.

### 2.4. Metabolite Alterations in Plasma Indicate Radiation-Induced GI Injury

**Figure 2:**
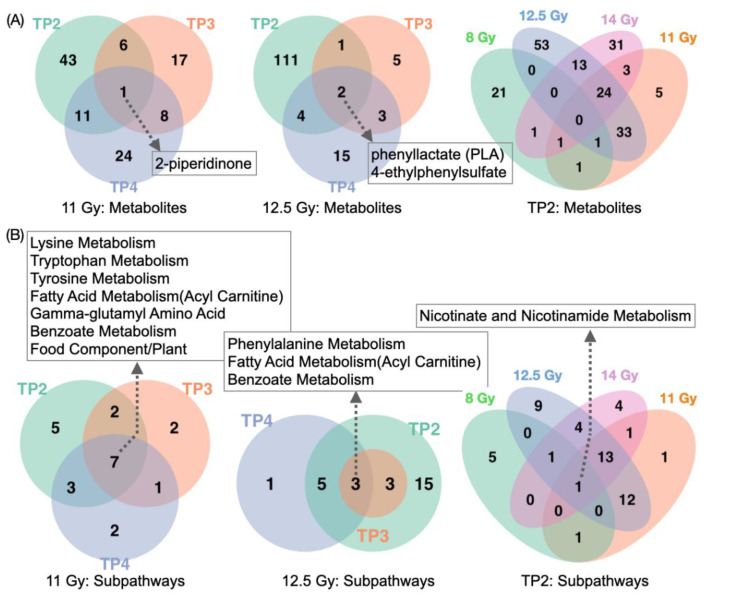
Venn diagrams highlighting common and distinct significantly dysregulated (**A**) metabolites and (**B**) subpathways, where metabolites and subpathways were excluded if they were also identified as markers for 0 Gy indicating non-radiation-induced markers. For both (**A**,**B**), comparisons between TP1 and TP2, TP3, TP4 and comparisons between 0 Gy and 8 Gy, 11 Gy, 12.5 Gy, 14 Gy were considered. The four statistical strategies (univariate analysis with *p* value < 0.05, fold change > 2, OPLS-DA with VIP values > 1, subpathway enrichment analysis with *p* value < 0.05) were applied to identify significantly dysregulated metabolites and subpathways.

We applied orthogonal partial least squares discriminant analysis (OPLS-DA) (variables importance for the projection [VIP] > 1) and subpathway enrichment analysis (hypergeometric *p* value < 0.05), which narrowed down the list of significantly dysregulated metabolites with a *p* value < 0.05 in Table 2. Figure 2 presents the number of common and distinct dysregulated metabolites (for more information in Table S1). To handle time varying confounders, metabolites, which were identified as differentially abundant between TP1 and TP2, TP1 and TP3, TP1 and TP4 for 0 Gy were manually filtered out for corresponding time point comparisons for 11 Gy and 12.5 Gy. First, the OPLS-DA results showed similar patterns to univariate analysis results in discriminant metabolites (data not shown). Second, 8 Gy showed different patterns in metabolite alterations from 11 Gy, 12.5 Gy, and 14 Gy. No single significant metabolite common in all radiation doses was identified. Third, only a couple of metabolites were identified as common in TP2, TP3, TP4 when comparing 11 Gy and 12.5 Gy with 0 Gy. Figure 2A indicates that the number of metabolites whose concentration changed in radiation dose- and time-dependent ways. Figure 2B represents the same information in Figure 2A, but at the subpathway level. Note that Venn diagrams before removing metabolites due to time varying confounders were included as Supplementary Materials (Figure S2). The number of dysregulated subpathways was increased when radiation dose increased from 11 Gy to 12.5 Gy. Interestingly, nicotinate and nicotinamide metabolism was identified as a significantly dysregulated subpathway for all radiation dose groups, where three metabolites (nicotinamide [niacin; vitamin B3], quinolinate, and N1-Methyl-2-pyridone-5-carboxamide) were responsible for the identification of the subpathway. Specifically, nicotinamide (down-regulated) and quinolinate (up-regulated) were identified for 8 Gy and 11 Gy, nicotinamide (down-regulated) and N1-Methyl-2-pyridone-5-carboxamide (up-regulated) for 12.5 Gy, quinolinate (up-regulated) and N1-Methyl-2-pyridone-5-carboxamide (up-regulated) for 14 Gy.

Quinolinate and nicotinamide are precursors of the coenzymes nicotinamide-adenine dinucleotide (NAD^+^) and its reduced form nicotinamide-adenine dinucleotide phosphate (NADH). As a precursor for NAD^+^, quinolinate can redirect tryptophan catabolism in order to replenish cellular NAD^+^ levels in response to inflammation and infection [7]. However, dysregulation of the kyrunenine pathway can lead to accumulation of quinolinate and can be a cause of inflammation through the increase of intermediate metabolites such as kynurenine, kynurenic acid, and quinolinate [8]. Nicotinamide has anti-inflammatory properties and plays important roles in host immunity [9]. Nicotinamide, a vitamin, is obtained from diet and can be also produced by the bacterial microflora in the intestines. Therefore, serious imbalance of the gut flora, which may occur after intestinal radiation damage, could lead to deficiency of this metabolite. Nicotinamide is the primary precursor of NAD^+^, an essential coenzyme for ATP that contributes to DNA repair and the sole substrate of the nuclear enzyme poly-ADP-ribose polymerase-1 (PARP-1)—an important enzyme in the DNA repair pathway [10,11]. Therefore, radiation-induced nicotinamide down-regulation may contribute to compromised DNA-repair and subsequent death of intestinal mucosal epithelial cells, leading to reduction in mucosal surface area, villus height, and crypt depth. Levels of nicotinamide concentration have been measured indirectly using the intermediary and end products of nicotinamide metabolism including N1-Methyl-2-pyridone-5-carboxamide. N1-Methyl-2-pyridone-5-carboxamide may accumulate under disease conditions resulting in accelerated DNA damage and retention of catabolic products [12].

**Figure 3:**
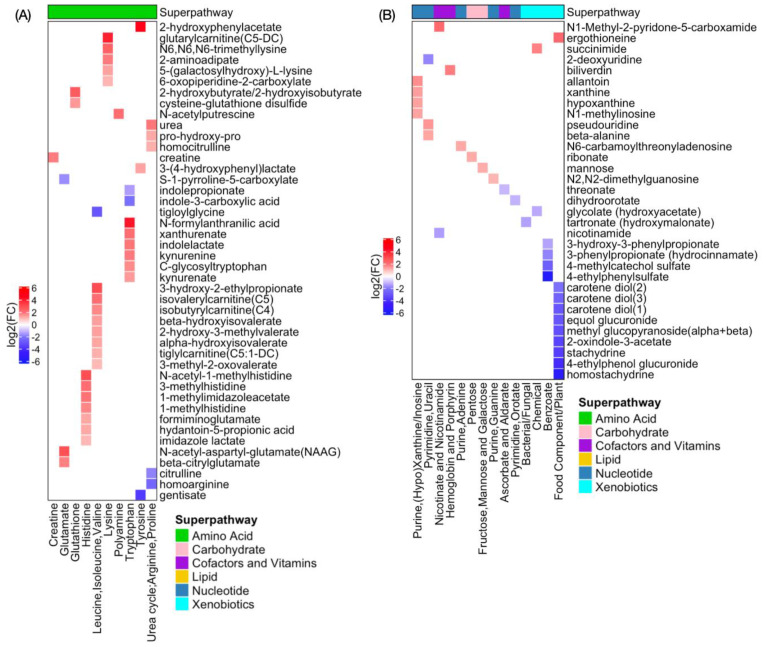

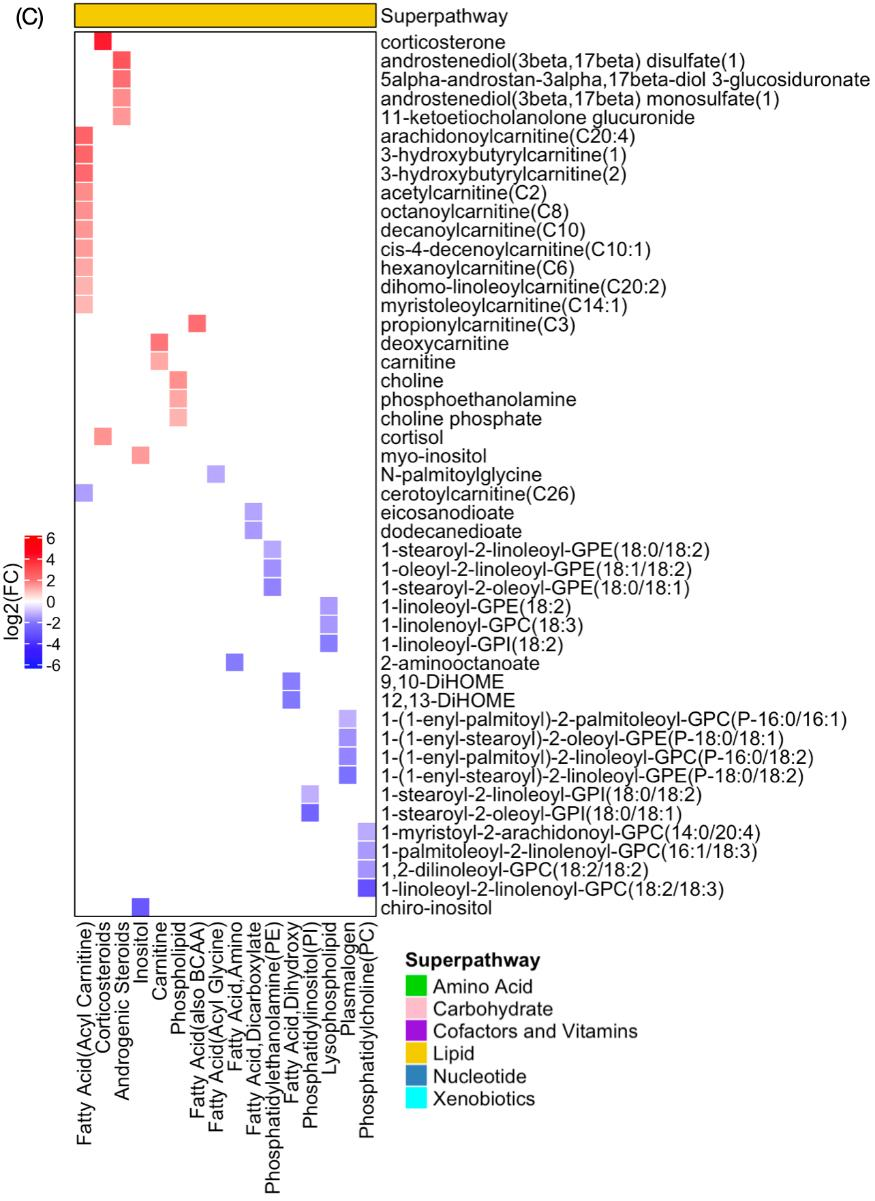
Heatmaps showing significantly dysregulated metabolites between 0 Gy and 12.5 Gy exposure at TP2 at the subpathway level for (**A**) amino acids, (**B**) carbohydrate, cofactors and vitamins, nucleotide, xenobiotics, and (**C**) lipid superpathway. The *x*-axis represents subpathways.

**Figure 4:**
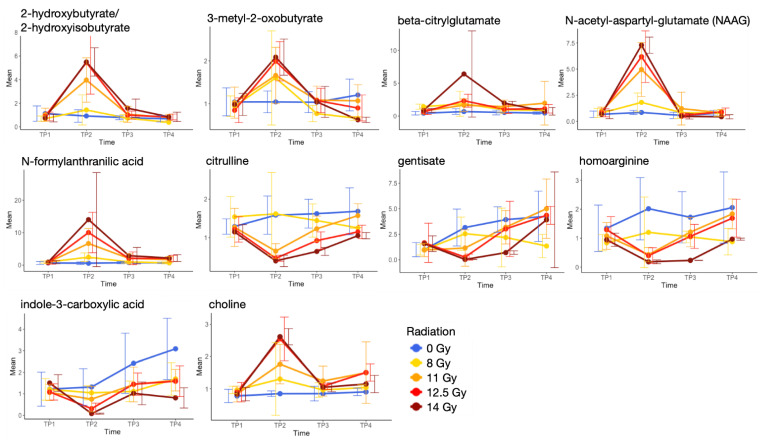
Metabolites in amino acid and lipid superpathways, which show changes according to the radiation dose level at TP2.

The largest number of metabolic changes compared to 0 Gy and TP1 occurred with a treatment of 12.5 Gy and at time point TP2, which might correlate with radiation-induced normal tissue toxicity. Figure 3 shows significantly up- (red) and down- (blue) regulated metabolites after 12.5 Gy at time point TP2 where the *x*-axis represents subpathways for each superpathway. First, there is elevation of many different amino acids and associated intermediates from their metabolic pathways, which may be a response to the increased polyamines and predicted increased cell growth needed to repair the intestinal lining. Increased urea might be a consequence of the gut inflammation and might indicate disruption or dysfunction of the intestinal barrier, which leads to the increased intestinal permeability [13,14]. Increased creatine and N-acetylputrescine could help the cell proliferation needed to repair the damaged intestinal lining. The elevated level of urea could be driving increased carbamoylation of lysine generating the elevated homocitrulline observed. Methylhistidines were also noted with increased concentration, which may indicate muscle breakdown or proteolysis. Dysregulated metabolites associated with tryptophan and indoles could reflect radiation-induced changes in the gut microbiome at 9 and 10 days after 12.5 Gy exposure [15]. Radiation caused a decrease in citrulline, a non-proteinogenic amino acid that has been identified as a biomarker for radiation-induced injury associated with GI ARS [16]. The decrease in citrulline suggests a decrease in enterocyte mass in the gut that might indicate potential benefit of citrulline supplementation on the radiation-induced gut toxicity. The concentration of citrulline was restored at TP3 and TP4, suggesting a recovery of the intestinal tissue (Figure 4). We also observed a decrease in citrulline at TP2 and its restoration at TP3 and TP4 with 11 Gy and 14 Gy. Low plasma concentrations of homoarginine are associated with an increased risk of cardiovascular events [17], and irradiation decreased homoarginine at TP2 with 12.5 Gy exposure. Gentisate has antioxidant and radioprotective properties and carries protection of the human erythrocytes against irradiation [18,19]. In our model, irradiation decreased gentisate levels. Indolepropionate is one of the serum metabolites reflecting gut microbiome alpha diversity in type 2 diabetes [20]. The reduced concentration of indolepropionate in our model might indicate a reduced alpha diversity due to radiation-induced changes in the gut microbiome. Most of the xenobiotic significant metabolites were down-regulated, whereas most of the metabolites in the categories of carbohydrates, cofactors and vitamins, and nucleotide metabolism were up-regulated (except for threonate, dihydroorotate, nicotinamide, and 2-deoxyuridine). The decrease in nicotinamide may suggest elevated lysine catabolism and excitotoxic activity [21], and thus dietary nicotinamide supplementation could mitigate radiation-induced injury [22]. As might be expected of animals with distressed GI tracts, there were indications that irradiated animals had different eating habits compared to the sham group. N-palmitoyl glycine, which is a novel endogenous lipid that acts as a modulator of calcium influx and nitric oxide production in sensory neurons [23], was decreased. Most of the fatty acid metabolism pathways were down-regulated. On the other hand, 3-hydyroxybutyrylcarnitine, which can be produced from either intermediate of fatty acid β-oxidation or ketone body metabolism in human skeletal muscle, was up-regulated. We speculate that it is likely related to the disruption of the intestinal lining by irradiation, that causes a change in nutrient absorption from the diet. This is consistent with the finding of a decrease in most xenobiotic metabolites. Both observations support a decrease in oral feeding derived nutrient uptake in irradiated animals. Decreases in lysophospholipids and plasmalogens may be the result of sloughing of intestinal cells directly into the intestine rather than through cell lysis and/or remodeling processes. Phosphatidylcholine- (PC), phosphatidylethanolamine- (PE), and phosphatidylinositol- (PI) derived lipids showed lower levels in plasma that could be associated with characteristics of unfavorable cardiometabolic risk profiles [24]. Irradiation also caused increases in multiple corticosteroids, cortisol and corticosterone, likely from physiological stress. Note that metabolites from energy metabolism pathways (including the tricarboxylic acid cycle), primary bile acid, and secondary bile acid did not pass our filtering steps. Choline is involved in osmoregulation and detoxification and may alleviate gastrointestinal injury [25]. We identified three significantly increased metabolites, choline, phosphoethanolamine, and choline phosphate, that were involved in phospholipid metabolism, but not trimethylamine N-oxide, which is generated in the host liver from trimethylamine and produced as a result of gut microbial metabolism of choline and could be associated with a radiation-induced cardiometabolic risk profile [24].

Among the metabolites involved in the amino acid and lipid superpathways in Figure 3, some showed changes according to the radiation dose level although those changes are not linear (Figure 4). These metabolites may be used for effective triage after unexpected irradiation.

## 3. Materials and Methods

### 3.1. NHP System

Two NHP cohorts (studies performed in 2016 and 2017) were combined in the study (Table 1). Animal housing, irradiation, and radiation dosimetry are described elsewhere [5]. In short, male and female adult animals were irradiated using a Clinac 21 EX 6 MV linear accelerator (Varian, Palo Alto, CA, USA) with a dose rate at mid-line of 0.75 ± 0.05 Gy/min in a field size of 20 × 20 cm, measuring inferiorly from the bottom of the last rib. Fifty percent of the dose was administered anteroposterior (AP) and 50% posteroanterior (PA). Animal care included monitoring environmental conditions, housing, diet and feed (PMI’s LabDiet^®^ Fiber-Plus^®^ Monkey Diet 5049), drinking water (provided ad libitum), and environmental enrichments (toys, soft toys, and a variety of appropriate treats). During in-life clinical observations, food consumption, and clinical pathology findings were recorded. All irradiated animals were administered the antibiotic enrofloxacin (5 mg/kg per day oral or intramuscular) on days 5 through 30 after irradiation and the antidiarrheal loperamide HCl (2 mg per day oral) on days 3 through 30. Analgesic support was provided on days 5 through 30 in the form of tramadol (25 mg per day oral) or buprenorphine (0.01 mg/kg intramuscular) if they did not want to take the tramadol. Lastly, animals received antiemetic ondansetron HCl (1.0 mg/kg intramuscular) as needed. An additional 4 animals (male) received sham irradiation for the 2016 cohort, and 3 animals (1 male/2 female) received sham irradiation over 60 days. The radiation groups consisted of sham (*n* = 7), 8 Gy (*n* = 12), 11 Gy (*n* = 24), 12.5 Gy (*n* = 12), and 14 Gy (*n* = 12). The 60 NHPs (in total) underwent the experimental protocol in block subsets of 10, balanced on day-dose (but not sex) to minimize variability in planned comparisons.

### 3.2. Plasma Sample Preparation and Instrumentation

Plasma samples were taken at various time points over a 60-day time course during the acclimation phase (D-5, D-1) and the post-exposure phase (Day 9, 10, 28, 29, 59, and 60 after exposure) from the 2 cohorts (2016, 2017), and maintained at −80 °C until processed. Samples were prepared using the automated MicroLab STAR^®^ system (Hamilton, Reno, NV, USA). To remove protein, dissociate small molecules bound to protein or trapped in the precipitated protein matrix, and recover chemically diverse metabolites, proteins were precipitated with methanol under vigorous shaking for 2 min (GenoGrinder 2000, Glen Mills, Clifton, NJ, USA) followed by centrifugation. The resulting extract was divided into 5 fractions: 2 for analysis by 2 separate reverse phase (RP) ultraperformance liquid chromatography-tandem mass spectrometry (UPLC-MS/MS) methods with positive ion mode electrospray ionization (ESI), one for analysis by RP-UPLC-MS/MS with negative ion mode ESI, one for analysis by hydrophilic interaction liquid chromatography (HILIC)/UPLC-MS/MS with negative ion mode ESI, and one sample was reserved for backup. Samples were placed briefly on a TurboVap^®^ (Zymark, Clackamas, OR, USA) to remove the organic solvent. The sample extracts were stored overnight under nitrogen before preparation for analysis. For global metabolomic profiling, all methods used Waters ACQUITY UPLC and Thermo Scientific Q-Exactive high resolution/accurate mass spectrometer interfaced with heated electrospray ionization (HESI-II) source and Orbitrap mass analyzer operated at 35,000 mass resolution. The sample extract was dried and then reconstituted in solvents. Each reconstitution solvent contained a series of standards at fixed concentrations to ensure injection and chromatographic consistency. The MS analysis alternated between MS and data-dependent MS^n−^ scans using dynamic exclusion. Raw data were extracted, the peaks identified, quality controlled, metabolite quantification, and data normalization processed using Metabolon’s in-house hardware and software.

### 3.3. Statistical Analyses

A total of 696 compounds of known identity for the 2 cohorts (2016, 2017) were detected in 200 plasma samples. Prior to any preprocess steps, normalization for plasma volume was performed. Then, we excluded 118 metabolites which were not detected in more than 20% of samples, followed by log transformation and imputation of missing values, if any with the minimum observed value for each compound were performed in the preprocessing of metabolomics data. We first examined global metabolomic profiles between the 2 cohorts for each time point and each radiation treatment that did not show significant differences in three-dimensional ordination space so that we combined the 2 cohorts to increase statistical power. Due to the complex nature of the study design (see Table 1), not all groups were powered well enough for a robust statistical analysis. Hence, the main comparisons were between the pre-exposure (TP1) and post-exposure (TP2, TP3, TP4) groupings for 0, 8 Gy, 11 Gy, 12.5 Gy and 14 Gy, and between sham (0 Gy) and irradiated groups (8 Gy, 11 Gy, 12.5 Gy and 14 Gy) for time point TP1, TP2, TP3, and TP4. Global metabolic profiles were determined from the experimental groups outlined in Table 1. For univariate analysis, we calculated fold change defined as the ratio of 2 means of a pair of groups of interest and performed two-sided Welch’s two-sample *t*-test to identify significantly dysregulated metabolites between experimental groups. Further, the BH multiple testing was applied to adjust the *p*-value from Welch’s *t*-test. A summary of the number of metabolites that achieved statistical significance (*p* < 0.05, or BH-adjusted *p*-value < 0.05) and fold change >2 is included in the analysis. For multivariate statistical analysis, we performed PCA to examine the effect of radiation doses and timepoints based on a qualitative visual inspection of the clustering pattern of plasma metabolomics profiles. Subsequently, OPLS-DA supervised statistical analysis was performed to identify metabolites responsible for the separation of the experimental groups. The quality of the OPLS-DA model was evaluated by R^2^ (goodness of fit, how well the model fits the data) and Q^2^ (goodness of prediction). Further, overfitting of the OPLS-DA model was validated by cross validated ANOVA and a permutation test. A highly significant *p*-value indicates the robustness and statistical significance of the model. In this study, we applied 10-fold cross validation if possible, otherwise 5-fold cross validation, depending on the number of samples, and the default number of permutations was 100. The VIP value of each metabolite in OPLS-DA models indicates its contribution to the classification. The VIP values of metabolites >1.0 were considered significant. To investigate the effect of dysregulated metabolites on subpathways, we calculated the *p*-value for significantly dysregulated (under- or over-enriched) metabolites involved in a given subpathway based on the cumulative distribution function of the hypergeometric distribution. Combining univariate analysis (fold change, *t*-test), OPLS-DA, and subpathway enrichment analysis, we finally selected metabolite biomarkers (fold change > 2, *t*-test *p*-value < 0.05, VIP > 1.0 from the validated OPLS-DA models) that belonged to the subpathways with at least 5 metabolites and subpathway *p*-value < 0.05 from hypergeometric test. All figures were generated using custom R scripts.

## 4. Conclusions

This study used Rhesus monkeys as a NHP model of abdominal irradiation. The two cohorts (and sham animals) irradiated at 0 Gy, 8 Gy, 11 Gy, 12.5 Gy, and 14 Gy exposure were combined, with statistical analysis of timepoints and treatment effects. Radiation exposure caused detectable metabolomic changes, especially for closest time-points before and after irradiation. In accordance with prior studies [16], this study identified citrulline, known as a biomarker for GI-ARS, with a decrease in concentration after irradiation and showing a dose-dependent response and recovery time. It also caused increases in multiple corticosteroids, likely from the physiological stress. The study identified significant changes in multiple metabolic pathways involving cell growth and GI tract health. There was evidence of increased fatty acid β-oxidation in the higher dose groups up to 9 and 10 days after radiation exposure. This is consistent with GI tract dysfunction caused by irradiation, leading to increased use of lipid stores for energy production. In addition, dysregulated metabolites, including tryptophan, indoles, and indolepropionate, which might indicate changes in the microbiome, were detected. This study of partial body irradiation focused on the GI tract in NHPs has generated a panel of metabolites that may serve not only as candidate plasma biomarkers for radiation injury in the GI tract, but also as a potential target for therapeutic intervention. Biomarkers that showed radiation dose-dependent changes could be used as quantifiable biomarkers for measurement of the presence and level of exposure in cases of unintentional irradiation and in radiation emergencies. However, this study lists metabolic changes but does not link them directly to the extent of GI damage in the same animals, for which further research is needed. Future studies need to include both males and females at sufficient numbers to identify metabolic profiles in each of the sexes with high power.

## Figures and Tables

**Table 1:** Number of plasma samples collected by radiation dose and pre- and post-irradiation days. Two cohorts were combined after examining global metabolomic profiles by ordination analysis that did not show much differences between cohorts. D-5 and D-1 were grouped into the group TP1 (pre-exposure), D9 and D10 into TP2, D28 and D29 into TP3, D59 and D60 into TP4 to power for a robust statistical analysis. Only male samples were included for downstream analysis after gender-specific patterns in the metabolomic data were observed.

Group	TP1 (Pre-Exposure)			TP2			TP3			TP4		
Day	D-5 ^a^	D-1 ^b^		D9 ^a^	D10 ^b^		D28 ^b^		D29 ^a^	D59 ^a^	D60 ^b^	
Sex	M	M	F	M	M	F	M	F	M	M	M	F
Sham	4	1	2	4	2	2	1	2	4	4	2	2
8 Gy	0	6	6	0	5	5	4	3	0	0	2	2
11 Gy	12	6	6	12	6	5	4	3	11	10	1	1
12.5 Gy	12	0	0	11	0	0	0	0	7	6	0	0
14 Gy	0	6	6	0	3	4	2	1	0	0	2	0

^a^ Samples collected in 2016, males only ^b^ Samples collected in 2017, males and females Abbreviations: D, day; F, female; M, male; TP, time point.

**Table 2:** Differential abundance analysis. The table represents the number of metabolites that showed significant changes for pairs of groups for radiation dose (**A**) and time point (**B**) where a fold change of 2 and *p* value < 0.05 were used as the cutoff. The numbers in parentheses represent the number of differentially abundant metabolites with a fold change > 2 and BH-adjusted *p* value < 0.05.

(**A**)							
**Radiation**		**TP1 vs. TP2**		**TP1 vs. TP3**		**TP1 vs. TP4**	
0 Gy		36 (0)		54 (0)		82 (6)	
8 Gy		12 (0)		9 (0)		25 (3)	
11 Gy		116 (112)		86 (70)		122 (116)	
12.5 Gy		173 (150)		58 (11)		94 (51)	
14 Gy		122 (29)		34 (5)		22 (0)	
(**B**)							
**Time**	**0 Gy vs. 8 Gy**		**0 Gy vs. 11 Gy**		**0 Gy vs. 12.5 Gy**		**0 Gy vs. 14 Gy**
TP1	7 (0)		6 (0)		4 (0)		2 (0)
TP2	37 (0)		92 (64)		154 (112)		128 (34)
TP3	18 (0)		25 (4)		23 (2)		51 (0)
TP4	43 (0)		13 (0)		26 (1)		54 (4)

## Data Availability

All data which support the findings of the study are within the manuscript and in Supplemental Materials.

## Supplementary Materials

The following are available online at https://www.mdpi.com/article/10.3390/metabo11080540/s1, Figure S1. PCA plots. (A) female, and (B) male samples, Figure S2. Venn diagrams highlighting common and distinct significantly dysregulated (A) metabolites and (B) subpathways before removing metabolites and subpathways to adjust for the time factor, Table S1. List of metabolites for intersections in Venn Diagrams in Figure 2A.

## References

[1] Waselenko J.K., MacVittie T.J., Blakely W.F., Pesik N., Wiley A.L., Dickerson W.E., Tsu H., Confer D.L., Coleman C.N., Seed T. (2004). Medical Management of the Acute Radiation Syndrome: Recommendations of the Strategic National Stockpile Radiation Working Group. Ann. Intern. Med..

[2] Dörr H., Meineke V. (2011). Acute radiation syndrome caused by accidental radiation exposure-therapeutic principles. BMC Med..

[3] Singh V., Olabisi A.O. (2017). Nonhuman primates as models for the discovery and development of radiation countermeasures. Expert Opin. Drug Discov..

[4] Singh V., Newman V.L., Berg A.N., MacVittie T.J. (2015). Animal models for acute radiation syndrome drug discovery. Expert Opin. Drug Discov..

[5] Wang J., Garg S., Landes R.D., Liu L., Fu Q., Seng J., Boerma M., Thrall K., Hauer-Jensen M., Pathak R. (2021). Differential Recovery of Small Intestinal Segments after Partial-Body Irradiation in Non-Human Primates. Radiat. Res..

[6] Trabado S., Al-Salameh A., Croixmarie V., Masson P., Corruble E., Fève B., Colle R., Ripoll L., Walther B., Boursier-Neyret C. (2017). The human plasma-metabolome: Reference values in 800 French healthy volunteers; impact of cholesterol, gender and age. PLoS ONE.

[7] Moffett J.R., Arun P., Puthillathu N., Vengilote R., Ives J.A., Badawy A.A.-B., Namboodiri A.M. (2020). Quinolinate as a Marker for Kynurenine Metabolite Formation and the Unresolved Question of NAD+ Synthesis During Inflammation and Infection. Front. Immunol..

[8] Raison C.L., Dantzer R., Kelley K.W., Lawson M.A., Woolwine B., Vogt G., Spivey J.R., Saito K., Miller A.H. (2009). CSF concentrations of brain tryptophan and kynurenines during immune stimulation with IFN-α: Relationship to CNS immune responses and depression. Mol. Psychiatry.

[9] Yoshii K., Hosomi K., Sawane K., Kunisawa J. (2019). Metabolism of Dietary and Microbial Vitamin B Family in the Regulation of Host Immunity. Front. Nutr..

[10] Oei S.L., Ziegler M. (2000). ATP for the DNA Ligation Step in Base Excision Repair Is Generated from Poly(ADP-ribose). J. Biol. Chem..

[11] Murata M.M., Kong X., Moncada E., Chen Y., Imamura H., Wang P., Berns M., Yokomori K., Digman M.A. (2019). NAD+ consumption by PARP1 in response to DNA damage triggers metabolic shift critical for damaged cell survival. Mol. Biol. Cell.

[12] Slominska E., Smolenski R., Szolkiewicz M., Leaver N., Rutkowski B., Simmonds H.A., Swierczynski J. (2002). Accumulation of plasma N-methyl-2-pyridone-5-carboxamide in patients with chronic renal failure. Mol. Cell. Biochem..

[13] Karl J.P., Margolis L., Madslien E.H., Murphy N.E., Castellani J., Gundersen Y., Hoke A.V., LeVangie M.W., Kumar R., Chakraborty N. (2017). Changes in intestinal microbiota composition and metabolism coincide with increased intestinal permeability in young adults under prolonged physiological stress. Am. J. Physiol. Liver Physiol..

[14] Lau W.L., Kalantar-Zadeh K., Vaziri N.D. (2015). The Gut as a Source of Inflammation in Chronic Kidney Disease. Nephron.

[15] Singh V.K., Newman V.L., Romaine P.L., Hauer-Jensen M., Pollard H.B. (2016). Use of biomarkers for assessing radiation injury and efficacy of countermeasures. Expert Rev. Mol. Diagn..

[16] Bujold K., Hauer-Jensen M., Donini O., Rumage A., Hartman D., Hendrickson H.P., Stamatopoulos J., Naraghi H., Pouliot M., Ascah A. (2016). Citrulline as a Biomarker for Gastrointestinal-Acute Radiation Syndrome: Species Differences and Experimental Condition Effects. Radiat. Res..

[17] Rodionov R.N., Oppici E., Martens-Lobenhoffer J., Jarzebska N., Brilloff S., Burdin D., Demyanov A., Kolouschek A., Leiper J., Maas R. (2016). A Novel Pathway for Metabolism of the Cardiovascular Risk Factor Homoarginine by alanine:glyoxylate aminotransferase 2. Sci. Rep..

[18] Joshi R., Gangabhagirathi R., Venu S., Adhikari S., Mukherjee T. (2011). Antioxidant activity and free radical scavenging reactions of gentisic acid: In-vitro and pulse radiolysis studies. Free. Radic. Res..

[19] Ashidate K., Kawamura M., Mimura D., Tohda H., Miyazaki S., Teramoto T., Yamamoto Y., Hirata Y. (2005). Gentisic acid, an aspirin metabolite, inhibits oxidation of low-density lipoprotein and the formation of cholesterol ester hydroperoxides in human plasma. Eur. J. Pharmacol..

[20] Menni C., Zhu J., Le Roy C.I., Mompeo O., Young K., Rebholz C.M., Selvin E., North K.E., Mohney R.P., Bell J.T. (2020). Serum metabolites reflecting gut microbiome alpha diversity predict type 2 diabetes. Gut Microbes.

[21] Lee Y., Khan A., Hong S., Jee S.H., Park Y.H. (2017). A metabolomic study on high-risk stroke patients determines low levels of serum lysine metabolites: A retrospective cohort study. Mol. BioSyst..

[22] Batra V., Kislay B. (2013). Mitigation of gamma-radiation induced abasic sites in genomic DNA by dietary nicotinamide supplementation: Metabolic up-regulation of NAD+ biosynthesis. Mutat. Res. Mol. Mech. Mutagen..

[23] Rimmerman N., Bradshaw H.B., Hughes H.V., Chen J.S.-C., Hu S.S.-J., McHugh D., Vefring E., Jahnsen J.A., Thompson E.L., Masuda K. (2008). N-Palmitoyl Glycine, a Novel Endogenous Lipid That Acts As a Modulator of Calcium Influx and Nitric Oxide Production in Sensory Neurons. Mol. Pharmacol..

[24] Roe A.J., Zhang S., Bhadelia R.A., Johnson E.J., Lichtenstein A.H., Rogers G.T., Rosenberg I.H., Smith C.E., Zeisel S.H., Scott T.M. (2017). Choline and its metabolites are differently associated with cardiometabolic risk factors, history of cardiovascular disease, and MRI-documented cerebrovascular disease in older adults. Am. J. Clin. Nutr..

[25] Ueland P.M. (2010). Choline and betaine in health and disease. J. Inherit. Metab. Dis..

