# Supplementary material for: Plasma Metabolomics in a Nonhuman Primate Model of Abdominal Radiation Exposure
Source: Metabolites. 2021 Aug 13;11(8):540. doi: 10.3390/metabo11080540 (PMC8398377; doi:10.3390/metabo11080540)
Supplement: Supplementary file 1 [file metabolites-11-00540-s001.zip › metabolites-1331054-supplementary.pdf]

metabolites

Article

Plasma Metabolomics in a Nonhuman Primate Model of Abdominal Radiation Exposure

Se-Ran Jun[1,*], Marjan Boerma[2], Zulema Udaondo[1], Sasha Richardson[3], Karla D. Thrall[4], Isabelle R. Miousse[5], John Seng[2], Rupak Pathak[2] and Martin Hauer-Jensen[2]

1 Department of Biomedical Informatics, University of Arkansas for Medical Sciences, Little Rock, AR 72205, USA; sjun@uams.edu (S.J.); zdominguez@uams.edu (Z.U.)
2 Division of Radiation Health, University of Arkansas for Medical Sciences, Little Rock, AR 72205, USA; mboerma@uams.edu (M.B.); jeseng@uams.edu (J.S.); rpathak@uams.edu (R.P.); mhjensen@uams.edu (M.H.)
3 Department of Computer Science, Fayetteville State University, Fayetteville, NC 28301, USA; sfrichardson9@gmail.com (S.R)
4. Altasciences Preclinical Seattle, Everett, WA 98203, USA; kthrall@altasciences.com (K.T)
5 Department of Biochemistry and Molecular Biology, University of Arkansas for Medical Sciences, Little Rock, AR 72205, USA; iracinemiousse@uams.edu (I.M.)
* Correspondence: sjun@uams.edu; Tel.:+1-501-686-6025

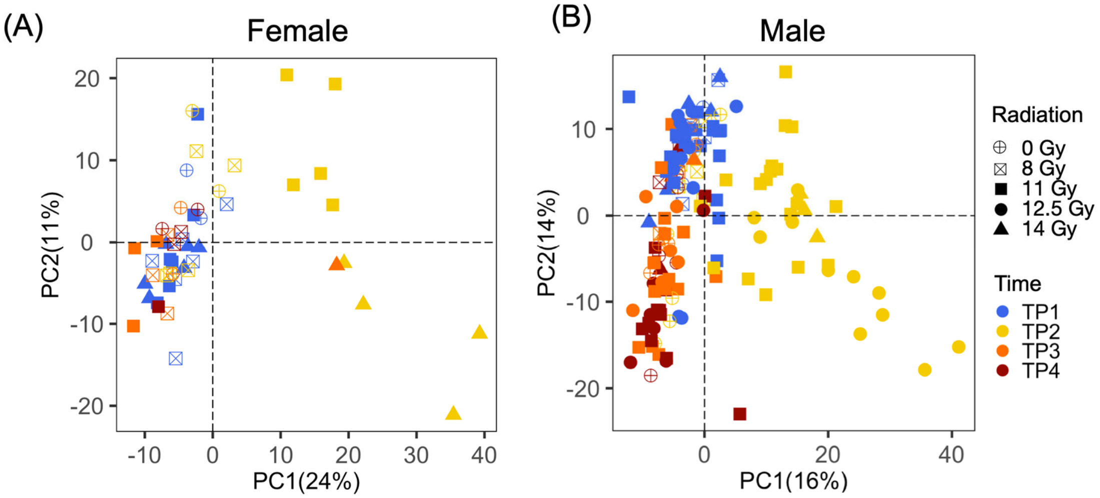

Figure S1. PCA plots. (A) female, and (B) male samples. Both male and female radiated samples were well separated from control samples in a similar way at time point TP2. Overall, plasma samples showed gender differences in metabolomics pattern in time points TP3 and TP4.

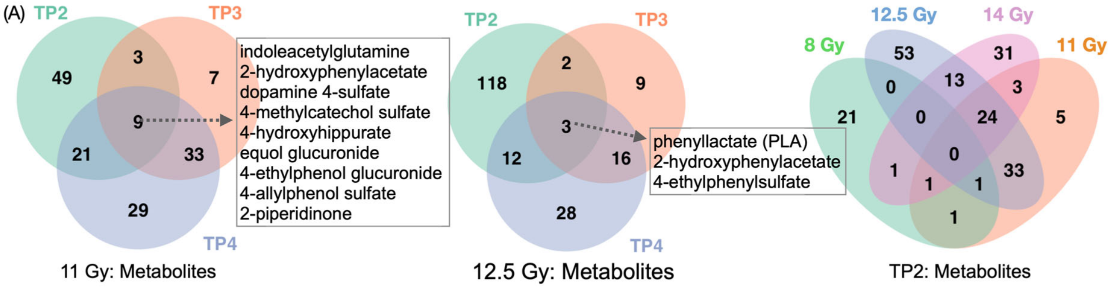

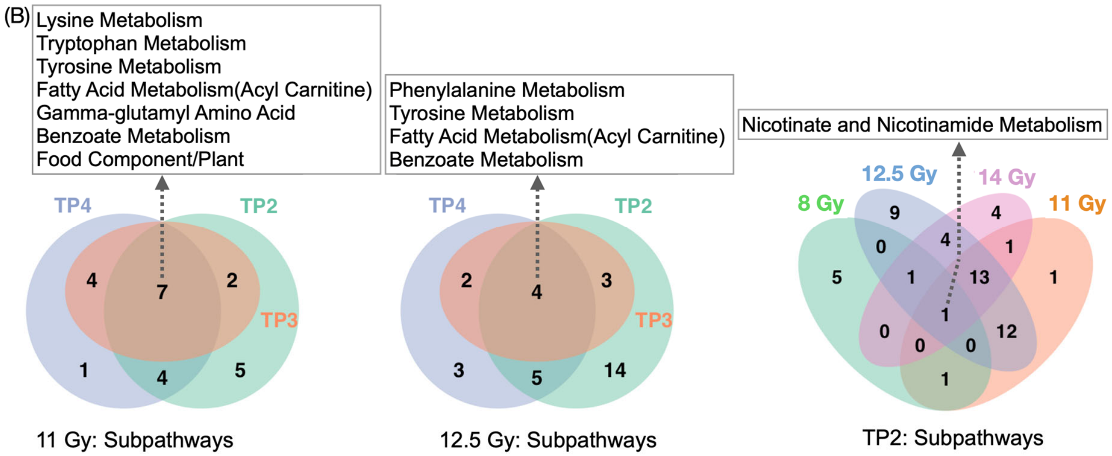

Figure S2. Venn diagrams highlighting common and distinct significantly dysregulated (A) metabolites and (B) subpathways before removing metabolites and subpathways to adjust time factor. For both (A) and (B), comparisons between TP1 and TP2, TP3, TP4 and comparisons between 0Gy and 8Gy, 11Gy, 12.5Gy, 14Gy were considered. The four statistical strategies (univariate analysis with p-value < 0.05, fold change greater than 2, OPLS-DA with VIP values greater than 1, subpathway enrichment analysis with p-value < 0.05) were applied to identity significantly dysregulated metabolites and subpathways.

Table S1. List of metabolites for intersections in Venn Diagrams in Figure 2(A).

Treatment	Intersection	Metabolite	Subpathway	Superpathway
11 Gy	TP1 vs TP2 and TP1 vs TP3	carboxyethyl-GABA	Glutamate Metabolism	Amino Acid
		N-acetylglutamine	Glutamate Metabolism	Amino Acid
		N-formylanthranilic acid	Tryptophan Metabolism	Amino Acid
		4-methylcatechol sulfate	Benzoate Metabolism	Xenobiotics
		equol glucuronide	Food Component/Plant	Xenobiotics
		4-ethylphenol glucuronide	Food Component/Plant	Xenobiotics
		2-piperidinone	Food Component/Plant	Xenobiotics
	TP1 vs TP2 and TP1 vs TP4	N-acetyl-1-methylhistidine*	Histidine Metabolism	Amino Acid
		hydantoin-5-propionic acid	Histidine Metabolism	Amino Acid
		3-methylglutaconate	Leucine, Isoleucine and Valine Metabolism	Amino Acid
		tigloylglycine	Leucine, Isoleucine and Valine Metabolism	Amino Acid
		N6,N6,N6-trimethyllysine	Lysine Metabolism	Amino Acid
		gamma-glutamyltyrosine	Gamma-glutamyl Amino Acid	Peptide
		gamma-glutamylthreonine	Gamma-glutamyl Amino Acid	Peptide
		4-ethylphenylsulfate	Benzoate Metabolism	Xenobiotics
		4-hydroxyhippurate	Benzoate Metabolism	Xenobiotics
		homostachydrine*	Food Component/Plant	Xenobiotics
		stachydrine	Food Component/Plant	Xenobiotics
		2-piperidinone	Food Component/Plant	Xenobiotics
	TP1 vs TP3 and TP1 vs TP4	5-aminovalerate	Lysine Metabolism	Amino Acid
		indoleacetylglutamine	Tryptophan Metabolism	Amino Acid
		cis-4-decenoylcarnitine (C10:1)	Fatty Acid Metabolism(Acyl Carnitine)	Lipid
		octanoylcarnitine (C8)	Fatty Acid Metabolism(Acyl Carnitine)	Lipid

		stearoylcarnitine (C18)	Fatty Acid Metabolism(Acyl Carnitine)	Lipid
		decanoylcarnitine (C10)	Fatty Acid Metabolism(Acyl Carnitine)	Lipid
		palmitoylcarnitine (C16)	Fatty Acid Metabolism(Acyl Carnitine)	Lipid
		3-hydroxyhippurate	Benzoate Metabolism	Xenobiotics
		2-piperidinone	Benzoate Metabolism	Xenobiotics
12.5 Gy	TP1 vs TP2 and TP1 vs TP	carboxyethyl-GABA	Glutamate Metabolism	Amino Acid
		phenyllactate (PLA)	Phenylalanine Metabolism	Amino Acid
		4-ethylphenylsulfate	Benzoate Metabolism	Xenobiotics
	TP1 vs TP2 and TP1 vs TP4	phenyllactate (PLA)	Phenylalanine Metabolisms	Amino Acid
		N-formylanthranilic acid	Tryptophan Metabolism	Amino Acid
		4-ethylphenylsulfate	Benzoate Metabolism	Xenobiotics
		homostachydrine*	Food Component/Plant	Xenobiotics
		erythritol	Food Component/Plant	Xenobiotics
		stachydrine	Food Component/Plant	Xenobiotics
	TP1 vs TP3 and TP1 vs TP4	phenyllactate (PLA)	Phenylalanine Metabolism	Amino Acid
		cis-4-decenoylcarnitine (C10:1)	Fatty Acid Metabolism(Acyl Carnitine)	Lipid
		octanoylcarnitine (C8)	Fatty Acid Metabolism(Acyl Carnitine)	Lipid
		decanoylcarnitine (C10)	Fatty Acid Metabolism(Acyl Carnitine)	Lipid
		4-ethylphenylsulfate	Benzoate Metabolism	Xenobiotics
